# SARS-COV-2 breakthrough infection and its covariates among healthcare providers of a hospital in Bangladesh during the omicron wave

**DOI:** 10.1016/j.heliyon.2024.e37287

**Published:** 2024-09-02

**Authors:** Masfida Akhter, Suman Kumar Roy, Abul Khair, Md Rabiul Karim, Ummel Khare Fatema Khan Mojlish, Minhaj Uddin Ahmed, Liaquat Ali

**Affiliations:** a Hamdard University Bangladesh, Bangladesh; b International Medical College and Hospital, Bangladesh; c Narayanganj 300 Bed Hospital, Bangladesh; d Pothikrit Institute of Health Studies, Bangladesh

**Keywords:** SARS-COV-2, COVID-19, Breakthrough infection, Health care workers, Anti-nucleocapsid antibody, Omicron

## Abstract

**Introduction:**

Breakthrough infection by SARS-COV-2 virus among vaccinated individuals has been reported from all over the world and it has created a substantial challenge in designing strategies to live with the virus in the post-pandemic era. Factors affecting the extent and nature of breakthrough infection are still not fully understood and those are known to vary depending on host and agent factors. Health Care Workers (HCWs), especially in hospital settings, are front-liners in combating the epidemic and, consequently, they are more vulnerable to breakthrough infection by SARS-COV-2. Like most of the countries of the world, Bangladesh went through several waves of COVID-19 and the last (3rd wave) was the widespread Omicron wave during the winter of 2022. HCWs in Bangladesh have been disproportionately affected by the virus. Under this context, the aim of the present study was to explore breakthrough infection (BTI) and its host-related covariates among HCWs of a COVID-dedicated city-based hospital during the Omicron wave in Bangladesh.

**Materials and methods:**

An observational cross-sectional study was conducted on 267 HCWs of the Narayanganj Tertiary (300-bed) hospital during February–March 2022 which coincided with the terminal part of the 3rd wave. Data were collected by trained Field Assistants using Interviewer-administered Data Collection Forms with Questionnaires as instruments. Previous COVID-19 status (any time after the onset of the pandemic and within last 3 months) was explored by the history of specific symptoms as well as by the confirmatory rtPCR test reports from DGHS approved laboratories Anti-nucleocapsid antibody (Anti-N-Ab) in venous blood samples, assayed by a chemiluminescent ELISA technique, was used as a seroprevalence-based marker of breakthrough infection during the preceding few months. Data were analyzed by bivariate as well as multivariate statistics using the IBM-SPSS software.

**Results:**

The median age (range) of the HCWs was 38 (21–65) years; Body Mass Index (BMI, kg/m^2^) 25 (15–49); and Waist-Hip Ratio (WHR) was 0.92 (0.46–1.21). The male subjects had significantly higher median age (p = 0.01) and higher WHR (p = 0.001) as compared to the female subjects. As per the BMI category, subjects with overweight and obesity constituted 83.3 % of the male subjects as compared to 61.6 % of the female subjects (p = 0.001). The time lapse between receiving of 3rd dose and blood sampling was significantly higher among females compared to males (median days 60 vs 49, p < 0.02) indicating earlier vaccination with 1st booster dose among females. A proportion of 51.85 % male and 49.68 % female subjects showed Anti-N-Ab positivity; there was no significant difference between the gender groups. Also, there was no significant difference among male and female subjects regarding the Ab levels. On Spearman correlation analysis, a tendency of association of WHR with Ab level was observed among the male subjects; however, the association did not show statistical significance (p = 0.09). On binary logistic regression Ab positivity was found to be independently associated with WHR (p = 0.03), and prior SARS-COV-2 infection within the last 3 months (p = 0.02) among males. When all the subjects were considered together, COVID symptom positivity during the last 3 months (p = 0.067) and receiving the 1st booster dose (p = 0.07) showed a tendency of association with Ab positivity. On multiple regression analysis, Ab levels showed a negative association with WHR (p = 0.035) among males.

**Conclusions:**

More than 50 % of the vaccinated hospital-based HCWs in Bangladesh suffered from BTI during the winter of 2022 when the Omicron wave (the 3rd wave) of COVID-19 was at its peak. The data also indicate that overweight and obesity are major host-related risk factors underlying BTI. Inadequate coverage by a booster dose seems to be another determinant of BTI and the level of immune response in this population.

## 1 Introduction

Breakthrough infection (BTI) by the SARS-COV-2 virus, defined as a fully vaccinated person getting infected with COVID-19 [1], has become an issue of major concern all over the world. Full vaccination in this case refers to the administration of two doses of effective vaccines following a recommended schedule and this has been generally adopted in the primary cycle of most of the COVID-19 vaccination programs. BTI has especially become a matter of concern with the emergence of the highly transmissible omicron variant of the virus which has a substantial capacity to evade natural as well as vaccine-mediated immunity through a number of subvariants. Apart from the clinical manifestations of the disorder, which may become serious in case of people with some comorbidities as well as with immunocompromised conditions, BTI may lead to widespread and continued mutation of the virus with the possibility of generation of a mutant with much more serious consequences [2].

While the existence of BTI has been confirmed in a number of populations, factors affecting the susceptibility to the infection as well as the accompanying immune response are not yet fully understood. Apart from factors related to ineffective vaccination, a number of risk factors have been suggested for BTI. Those include obesity as well as comorbidities like hypertension [3], diabetes [4], chronic kidney disease [5], lung diseases [6] and cancer [7]. In spite of the progress already made, understanding of the factors in individual racial, environmental and social settings are essential for devising long-term strategies to combat COVID-19 [8] which is now generally thought to persist in future as an endemic disease.

Assessment of BTI and its risk factors are particularly complicated in societies where periodic health check-up is not mandatory and voluntary testing for COVID-19 in due time is not practiced. Also, due to the asymptomatic or mildly symptomatic nature of omicron-induced infection in most cases, initiatives for testing are not common in these societies. Even the confirmatory test rtPCR may not be reliable if it is not within the right window of the infection cycle. The interpretation of the seroprevalence of anti-spike protein antibody, as a marker of the previous SARS-COV-2 infection, is complicated among vaccinated individuals since the major vaccines against COVID-19 specifically generates antibodies against spike proteins. One alternate possibility is to use anti-nucleocapsid antibody (Anti-N-Ab) for the estimation of seroprevalence. Natural infection generates antibodies against various other proteins of the virus including nucleocapsid protein (N-protein). Measurement of Anti-N-Ab, accordingly, may help in detecting breakthrough infection in recent months through estimation of seroprevalence [9] and it may be used to monitor natural infection after vaccine rollout [10,11].

The Health Care Workers (HCWs) of all categories are front-liners in combating the epidemic and, consequently, are more vulnerable to Covid-19. Accordingly, assessment of Anti-N-Ab in HCPs, with documented records of SARS-COV-2 infection and also with different levels of vaccination, may help in expanding our knowledge on the nature of breakthrough infection in this disease. In the present study, we investigated a group of HCWs during February–March 2022 in a tertiary care public hospital (Narayanganj 300-bed District Hospital) in Bangladesh located in a City adjacent to Dhaka, the capital of the country. The timing coincides with the terminal stage of the peak of the 3rd wave which has been reported to be caused almost exclusively by the Omicron variant of the virus [12]. Narayanganj was one of the earliest hotspots in the country and the hospital was declared a COVID-dedicated hospital since the beginning of the pandemic. Accordingly, the infection status (confirmed by rtPCR test) of the HCWs from this hospital was available from the early period of the pandemic. Also, as in the whole country, the HCWs were entitled to receive priority-based vaccine (including the 1st booster dose, ie 3rd dose) since its government-sponsored availability from the beginning of 2021. Following the national guideline (in line with the WHO suggestions) the primary full cycle with two doses of the vaccine (first one with Covisheild - an Oxford-AstraZeneca brand and the second one with Pfizer/Moderna in most cases) were received by almost hundred percent of the HCWs. The 1st booster dose, however, were received by only about one-third of the employees due to varying individual initiatives. This created a possibility to investigate the booster dose-related risk analysis in addition to the host-related risk factors of BTI among these HCWs.

Under the above context, the aim of the present study was to explore breakthrough infection (BTI) and its host-related covariates among HCWs of a COVID-dedicated city-based hospital during the Omicron wave in Bangladesh.

## 2 Materials and methods

The present cross-sectional single center study was conducted during February–March 2022 among 267 vaccinated HCWs (various categories) of the Narayanganj District Hospital. The study was approved by the Ethical Review Committee of the Pothikrit Institute of Health Studies [Ref: PIHS/ IRRB/ LOA/ 12(3); Date: 31 August 2021]. Written Consent was obtained by all participants. Patients hospitalized for reasons other than COVID-19 and HCWs refusing to participate were excluded from the present study. No HCW was required to be excluded for such reasons.

Data were collected by trained Research Assistants using Interviewer-administered standardized Data Collection Forms with Questionnaires (DCFQ) as the instrument. Divided into various sections, the DCFQ contained host factor-related information which included demographic (age), anthropometric [Body Mass Index (BMI) and Waist-Hip Ratio (WHR)], and clinical as well as behavioral factors which are relevant to BTI. Among the clinical factors there were COVID-19 infection-related variables including COVID-19 symptoms any time after the beginning of the pandemic (Covid symptom anytime + ve or –ve); COVID-19 in the three months preceding the study (Covid symptoms last 3 months + ve or –ve); COVID-19 rtPCR tests any time after the beginning of pandemic (Covid rtPCR anytime + ve or –ve); COVID-19 rtPCR test during the last 3 months (Covid rtPCR last 3 months + ve or –ve); Comorbidity [Diabetes, Hypertension, Chronic Obstructive Pulmonary Disease or COPD, Chronic Kidney Disease or CKD, Cancer, any other major chronic disease, Any of the earlier diseases (Any Comorbidity)]; and need for hospitalization and/or oxygen supplementation. Vaccine-related information was also recorded in a separate section where the number of doses, the brand of the vaccine for each specific dose and the date of receiving the doses were noted. The Body Mass Index (BMI) and Waist-Hip Ratio (WHR) were measured by standard formula and BMI was categorized as suggested for South-East Asian populations (Underweight, <18.5; Normal Weight, 18.5–22.9; Overweight, 23.0–27.5; Obese, ≥27.5).

Previous COVID-19 status (any time after the onset of the pandemic and within last 3 months) was explored by the history of specific symptoms as well as by the confirmatory rtPCR test reports from laboratories approved by the Director General of Health Services (DGHS). Antibody measurement was conducted using venous blood sample drawn through venepuncture by a trained laboratory technician. Serum was stored at 2–8 °C for semiquantitative SARS-COV-2 antibody measurement using the SARS-CoV-2 IgG Kit (REF: 6R86-22) manufactured by Abbott (USA) for its Architect Autoanalyzer. The automated two-step immunoassay uses a chemiluminescent microparticle immunoassay (CMIA) technology. The chemiluminescent reaction is measured as a relative light unit (RLU) and there is a direct relationship between the amount of IgG antibodies to SARS-CoV-2 in the sample and the RLU detected by the system optics. Results are reported by dividing the sample result by the stored calibrator result. The default result unit for the SARS-CoV-2 IgG assay is Index (S/C) and an Index equal to or above the cut-off value of 1.4 is interpreted as Ab-positive. The S/C Index was used as a surrogate marker of the Anti-N-Ab level.

Data analysis was performed by using the SPSS statistical package, v 26.0. For qualitative immunity status Anti-N-Ab positivity and for quantitative analysis, the S/C Index were the Dependent Variables respectively. Hospital admission and Need for Oxygen support were considered as markers of severity during the past infection. Descriptive analysis was conducted to determine the proportions of different categorical variables and the difference between proportions was statistically assessed by the Chi-square test. Continuous variables were expressed as mean (± standard deviation) or median (range). Comparison between Groups was done by appropriate parametric or nonparametric tests depending on the distribution of the data. The independent association of antibody positivity and Anti-N-Ab level with potential confounders was analyzed by binary logistic and multivariable linear regression analysis, respectively. A two-sided p-value <0.05 was considered as statistically significant.

## 3 Results

The median age (range) of the HCWs was 38 (21–65) years; BMI (kg/m^2^) 25 (15–49); and WHR was 0.92 (0.46–1.21). The male subjects had significantly higher median age (p = 0.01) and higher WHR (p = 0.001) as compared to the female subjects (Table 1). Based on BMI categories, overweight and obese subjects constituted 83.3 % of the male subjects as compared to 61.6 % of the female subjects (p = 0.001).

**Table 1:** Gender-wise characteristics and infection as well as vaccination status of the subjects.

Variable	Male, N = 108 (n%)	Female, N = 159 (n%)	Total, N = 267 (n%)	p-value (Male vs Female)
Age, year, Median (Range)	45 (25–60)	35 (21–65)	38 (21–65)	0.01
Age <40 years	51 (47.2 %)	93 (58.5 %)	144 (53.9 %)	0.21
Age 40–50 years	21 (19.4 %)	27 (17 %)	48 (18 %)	
Age >50 years	36 (33.3 %)	39 (24.5)	75 (28.1 %)	
BMI, kg/m^2^, Median (Range)	25 (15–49)	25 (15–41)	25 (15–49)	0.823
Low, BMI <18.5	5 (4.6 %)	16 (10.1 %)	21 (7.9 %)	0.001
Ideal, BMI 18.5–22.9	13 (12 %)	45 (28.3 %)	58 (21.7 %)	
Overweight, BMI 23.0–27.5	50 (46.3 %)	62 (39 %)	112 (41.9 %)	
Obese, BMI >27.5	40 (37.0 %)	36 (22.6 %)	76 (28.5 %)	
WHR, Median (Range)	0.92 (0.68–1.21)	0.91 (0.46–1.08)	0.92 (0.46–1.21)	<0.001
Low WHR (<0.95, M; <0.80, F)	61 (56.5 %)	22 (13.8 %)	83 (31.1 %)	0.161
Normal WHR (0.96–1.0, M; 0.81–0.85, F)	14 (13 %)	29 (18.2 %)	43 (16.1 %)	
High (WHR >1.0, M; >0.85, F	33 (30.6 %)	108 (67.9 %)	141 (52.8 %)	
Days after Dose 1 Median (Range)	360 (63–420)	270 (58–420)	360 (180–401)	0.025
Days after Dose 2 Median (Range)	270 (58–395)	210 (13–395)	300 (120–395)	0.05
Vaccine Dose 3 Status	38 (33.9 %)	49 (28,5 %)	90 (31.7 %)	0.51
Days after Dose 3 Median (Range)	49 (2–89)	60 (2–150)	52.5 (1–115)	0.02
RT-PCR positivity	97 (89.8 %)	129 (81.1 %)	226 (84.6 %)	0.76
Need for Hospitalization	27 (25 %)	32 (20.1 %)	59 (22.1 %)	0.37
Need for supplementary oxygen	6 (5.6 %)	11 (6.9 %)	17 (6.4 %)	0.80
Comorbidity	34 (31.5 %)	59 (37.1 %)	93 (34.8 %)	0.36

Data were expressed as number (percentage) [n (%)] or median (range) as appropriate. Chi-square and Mann-Whitney tests were used to find out the association between categorical and continuous variables, respectively. P < 0.05 was considered as significant. BMI, Body Mass Index; WHR, Waist-Hip Ratio.

Almost all the HCWs (96.85 %) were vaccinated with the 1st dose of COVID-19 vaccine; the 2nd dose was also received by 93.94 % HCWs. The 3rd dose, however, was received by only about one-third (31.66 %) subjects. Among the vaccinated, the majority of the HCWs (70.4 %) received the Oxford-AstraZeneca brand of COVID-19 vaccine (manufactured by Serum Institute of India) as the 1st dose; this was followed by Moderna (17.6 %) and Pfizer BioNTech (12.0 %). Regarding the 2nd dose 70.8 % HCWs received the Oxford-AstraZeneca, 17.6 % received Moderna and 11.6 % received the Pfizer BioNTech vaccines. Among the HCWs receiving the 3rd Dose 79.7 % were vaccinated by Pfizer BioNTech, 12.3 % by Oxford-AstraZeneca and 8.0 % were vaccinated by Moderna vaccines. All these brands were mRNA-based vaccines and no HCW received any inactivated vaccines for any of the doses.

Compared to male counterparts, female subjects were delayed in receiving the first and second doses; however, time lapse between receiving of 3rd dose and blood sampling in the present study was significantly higher among females compared to males (median days 60 vs 49, p < 0.02) indicating earlier vaccination with 1st booster dose among females.

As compared to 51.85 % of males, a proportion of 49.68 % of female subjects showed Anti-N-Ab positivity; there was no statistically significant difference between the male and female groups (Table 2). Also, there was no significant difference between male and female subjects regarding the Ab levels (Table 2).

**Table 2:** Anti-N-Ab status among male and female subjects.

Anti-N-Ab status	Male, n = 108 (n%)	Female, n = 159 (n%)	Total, n = 267 (n%)	p-value (Male vs Female)
Ab positivity	56 (51.85 %)	79 (49.68 %)	135 (50.56 %)	0.80
Ab level, S/C Index Median (Range)	1.75 (0–10.97)	1.28 (0–11.28)	1.46 (0–11.28)	0.63

Data were expressed as number (percentage) [n (%)] or median (range) as appropriate. Chi-square and Mann-Whitney tests were used to find out the association between categorical and continuous variables, respectively. P < 0.05 was considered as significant.

The risk factors for BTI were explored by comparative analysis between Anti-N-Ab positive and Anti-N-Ab negative subjects (Table 3). On Chi-square analysis, only COVID-19 symptom positivity during the last 3 months was found to be significantly associated with Ant-N-Ab positivity among males (p = 0.02) as well as among total subjects (p = 0.04). Among the female subgroup, the association was just outside the borderline of statistical significance (p = 0.05). Other factors (as shown in Table 3) like age, BMI, WHR, COVID-19 symptom positivity anytime, COVID-19 rtPCR positivity (anytime or during the last 3 months), Comorbidity (anytime, diabetes and hypertension) did not show any significant statistical difference between the Ant-N-Ab positive and Anti-N-Ab Negative subgroups as explored by appropriate tests. Additional factors like other comorbidities (COPD, CKD, cancer and any other chronic disease) and need for hospitalization and/ or oxygen supplementation were also analyzed (data not shown) and no significant difference was found between the Ab positive and negative subgroups among male, female or total subjects.

**Table 3:** Comparison of risk factors between Anti-N-Ab+ and Ant-N-Ab- subgroups among male, female and total subjects.

Variables	Male, n = 108 n (%)		p-value	Female, n = 159 n (%)		p value	Total,n = 267 n (%)		p-value
	Ab + ve (n = 56)	Ab-ve (n = 52)		Ab + ve (n = 79)	Ab-ve (n = 80)		Ab + ve (n = 135)	Ab-ve (n = 132)	
Age (in years), Median (Range)	42 (23–60)	40 (21–60)	0.51	34 (23–60)	35 (23–65)	0.61	38 (23–60)	37 (21–65)	0.40
*Age (Category)*									
21–40	25 (44.64)	26 (50)	0.83	45 (56.96)	48 (60)	0.14	70 (51.85)	74 (56.06)	0.21
40–50	11(19.64)	10 (19.23)		10 (12.65)	17 (21.25)		21 (15.55)	27 (20.45)	
>50	20 (35.71)	16 (30.76)		24 (30.37)	15 (18.75)		44 (32.59)	31 (23.48)	
BMI, Median (Range)	26 (15–39)	26 (16–41)	0.30	24 (16–42)	24 (15–49)	0.57	25 (15–42)	25 (15–49)	0.45
*BMI category*									
<18.5	2 (3.57)	3 (5.76)	0.83	9 (11.39)	7 (8.75)	0.70	11 (8.14)	10 (7.57)	0.84
<18.5–22.9	8 (14.28)	5 (9.61)		24 (30.37)	21 (26.25)		32 (23.70)	26 (19.69)	
23–27.5	25 (44.64)	25 (48.07)		31 (39.24)	31 (38.75)		56 (41.48)	56 (42.42)	
>27.5	21 (37.5)	19 (36.53)		15 (18.98)	21 (26.25)		36 (26.66)	40 (30.30)	
WHR, Median (Range)	0.94 (0.74–1.17)	0.96 (0.82–1.13)	0.07	0.89 (0.67–1.21)	0.90 (0.68–1.06)	0.74	0.91 (0.46–1.21)	0.92 (0.68–1.13)	0.27
*WHR category*									
Low WHR (<0.95, M; <0.80, F)	36 (64.28)	25 (48.07)	0.10	12 (15.18)	10 (12.5)	0.88	48 (35.55)	35 (26.51)	0.42
Normal WHR (0.96–1.0, M; 0.81–0.85, F)	8 (14.28)	6 (11.53)		14 (17.72)	15 (18.75)		22 (16.29)	21(15.90)	
High (WHR >1.0, M; >0.85, F	12 (21.42)	21(40.38)		53 (67.08)	55 (68.7)		65 (48.14	76 (57.57)	
COVID symptom anytime + ve	44 (78.57)	35 (67.31)	0.20	42 (53.16)	37 (46.25)	0.75	86 (63.7)	49 (37.12)	0.62
Covid Symptom anytime -ve	12 (21.43)	17 (32.69)		45 (56.96)	35 (43.75)		80 (59.26)	52 (39.39)	
Covid Symptom last 3 months + ve	9 (16.07)	47 (90.38)	0.02	19 (24.05)	60 (75)	0.05	28 (20.78)	107 (81.06)	0.04
Covid Symptom last 3 months -ve	1 (1.78)	51 (9.07)		15 (18.98)	65 (81.25)		16 (11.85)	116 (87.87)	
Covid rtPCR anytime + ve	20 (35.71)	36 (69.23)	0.84	26 (32.91)	53 (66.25)	0.22	46 (34.07)	89 (67.42)	0.24
Covid rtPCR anytime -ve	17 (30.36)	35 (67.31)		19 (24.05)	61 (76.25)		36 (26.66)	96 (72.72)	
Covid rtPCR last 3 months + ve	17 (30.35)	39 (75.00)	0.83	6 (7.59)	73 (91.25)	0.32	10 (7.41)	125 (94.69)	0.17
Covid rtPCR last 3 months -ve	14 (25.00)	38 (73.00)		3 (3.79)	77 (96.25)		4 (2.69)	128 (96.96)	
Any Comorbidity + ve	15 (26.78)	41 (78.85)	0.31	31 (39.24)	48 (60)	0.62	46 (34.07)	89 (67.42)	0.79
Any Comorbidity -ve	19 (32.93)	33 (63.46)		28 (35.44)	52 (65)		47 (34.81)	85 (64.39)	
Diabetes + ve	5 (8.93)	51 (98.08)	0.17	14 (17.72)	65 (81.25)	0.84	19 (14.07)	116 (87.87)	0.50
Diabetes -ve	10 (17.86)	42 (80.76)		13 (16.45)	67 (83.75)		23 (17.04)	109 (82.57)	
Hypertension + ve	7 (12.5)	49 (94.23)	0.30	17 (21.52)	62 (77.5)	0.55	24 (17.77)	111 (84.09)	0.88
Hypertension -ve	11 (19.64)	41 (78.85)		14 (17.72)	66 (82.5)		25 (85.52)	107 (81.06)	

Data were expressed as n (%) or median (range) as appropriate. Chi-square and Mann-Whitney tests were used to find out the association between categorical and continuous variables, respectively. P < 0.05 was considered as significant.

On Spearman correlation analysis, no significant correlation of Ab levels was found with any other variables either among male or female subjects; however, a tendency of association (not reaching statistical significance) of WHR with Ab level (p = 0.09) was observed only among the male subjects (Table 4).

**Table 4:** Correlation of Ab levels with age, BMI, WHR and time-lapse (in Days) after vaccination among male and female subjects.

Variables	Male (n = 108)		Female(n = 159)		Total (n = 267)	
	r	p	r	p	r	p
Age	0.124	0.202	0.017	0.828	0.014	0.50
BMI	−0.042	0.667	−0.071	0.372	−0.040	0.51
WHR	−0.164	0.090	−0.006	0.937	−0.028	0.65
Days after Dose1 vaccine	−0.024	0.806	−0.005	0.946	−0.007	0.90
Days after Dose2 vaccine	0.030	0.758	0.025	0.750	0.019	0.75
Days after Dose 3 vaccine	−0.023	0.812	−0.092	0.248	−0.014	0.82

BMI, Body mass index; WHR, Waist-Hip Ratio; Analysis was done by Spearman Correlation test; p < 0.05 was considered as statistically significant.

On binary logistic regression Ab positivity was found to be independently associated with WHR [OR (95 % CI), 1.17 (2.29–6.01); p = 0.026], and prior Covid infection within last 3 months [OR (95 % CI), 15.24 (1.54–151.00); p = 0.020] among males; however, no such association was found among the females (Table 5). When all the subjects were considered together, Covid symptom positivity during last 3-months (p = 0.067) and receiving the 1st booster dose (p = 0.07) showed a tendency of association with Ab positivity (Table 5).

**Table 5:** Binary logistic regression analysis with Ab positivity as dependent variable and potential confounders as independent variables.

Variables	Male		Female		Total	
	OR (95 % CI)	p	OR (95 % CI)	p	OR (95 % CI)	p
Age, year	0.97(0.92–1.01)	0.138	1.00(0.97–1.04)	0.957	0.98(0.96–1.01)	0.224
BMI	1.08(0.98–1.17)	0.125	1.03(0.96–1.10)	0.410	1.03 (0 0.98–1.09)	0.196
Waist hip ratio	1.17(2.29–6.01)	0.026	0.64(0.01–49.55)	0.838	7.03(0.33–150.95)	0.212
Comorbidity	0.46(0.15–1.41)	0.175	1.13(0.55–2.66)	0.630	0.79(0.43–1.46)	0.458
COVID symptom positivity anytime	2.15(0.73–6.35)	0.167	0.85(0.42–1.72)	0.643	1.21(0.69–2.10)	0.507
COVID report status anytime	0.63(0.23–1.71)	0.365	1.81(0.81–4.04)	0.147	1.21(0.67–2.20)	0.528
Previous 3-month COVID symptom positivity	15.24(1.54–151)	0.020	1.28(0.54–3.31)	0.578	1.88(0.92–3.83)	0.083
Previous 3-month COVID report status	2.26(0.22–23.56)	0.496	1.80(0.38–8.43)	0.457	1.89(0.54–6.59)	0.317
Days after Dose1 vaccine	1.00(0.99–1.01)	0.323	1.00(0.99–1.00)	0.346	1.00(0.99–1.00)	0.503
Days after Dose2 vaccine	0.99(0.99–1.01)	0.526	1.00(0.99–1.00)	0.719	1.00(0.99–1.00)	0.586
Days after Dose 3 vaccine	0.98(0.95–1.10)	0.185	0.99(0.97–1.02)	0.578	0.987 (0.97–1.00)	0.136
Vaccine Dose 3 Received	0.45(0.09–2.22)	0.324	0.43(0.12–1.54)	0.195	0.415(0.16–1.08)	0.071
Constant	0.000	0.030	0.516	0.827	0.101	0.324

Data were analyzed by Binary logistic regression analysis. Antibody positivity was considered as the dependent variable; OR, Odds Ratio; 95 % CI (95 % Confidence Interval); p < 0.05 was considered as statistically significant.

On multivariable linear regression analysis, the Ab level showed a negative association with WHR [RC (95 % CI), −0.22 (−19.01 to −0.74); (p = 0.035)] among males on adjustment of the effects of confounding variables (Table 6). Among females the Ab level did not show independent positive association with any other covariates.

**Table 6:** Independent association of Anti-N-Ab level on adjustment of confounding variables.

Variables	Male		Female		Total	
	RC (95 % CI)	p value	RC (95 % CI)	p value	RC (95 % CI)	p value
Age, year	0.13 (−0.03–0.12)	0.251	−0.08 (−0.09–0.04)	0.467	0.05(-0.03–0.07)	0.537
BMI	−0.04 (−0.19–0.12)	0.681	−0.07 (−0.17–0.07)	0.415	−0.05(-0.13–0.05)	0.415
WHR	−0.22(-19.01–−0.74)	0.034	0.07 (−4.73–11.16)	0.425	−0.04(-7.35–3.86)	0.539
Comorbidity	0.05 (−1.45–2.35)	0.639	−0.12 (−2.35–0.51)	0.206	−0.03 (−1.31–0.93)	0.732
Covid Symptom Positivity any time	−0.19 (−3.54–0.14)	0.070	1.00(-0.57–2.01)	0.270	−0.04 (−1.34–0.70)	0.541
Covid Symptom Positivity during last 3 months	−0.11(-4.15–1.30)	0.302	0.06 (−1.01–2.12)	0.487	−0.01 (−1.39–1.18)	0.870
Covid rtPCR test positivity, anytime	0.07(-1.14–2.33)	0.499	−0.06 (−1.90–0.99)	0.540	−0.01(-1.18–1.02)	0.886
Covid rtPCR test positivity during last 3 months	−0.01 (−3.84–3.46)	0.918	−0.16 (−5.23–0.18)	0.067	−0.10(-3.75–0.50)	0.133
Days after Dose1 vaccine	−0.14 (−0.02–0.01)	0.342	−0.02 (−0.01–0.01)	0.837	−0.03 (−0.01–0.01)	0.751
Days after Dose2 vaccine	0.08 (−0.01–0.02)	0.606	0.08 (−0.01–0.01)	0.515	0.06(-0.01–0.01)	0.483
Days after Dose 3 vaccine	0.19 (−0.02–0.08)	0.272	−0.03 (−0.05–0.04)	0.832	0.10(-0.02–0.05)	0.355
Vaccine Dose 3 Received	0.17 (−1.36–4.07)	0 0.325	0.11 (−1.47–3.10)	0.481	0.164(-0.41–3.01)	0.137
Constant	−1.84–29.90	0.082	−4.67–16.56	0.270	−1.14–15.06	0.092

Data were analyzed by multivariable linear regression analysis. Anti-N-Ab level was considered as the dependent variable; RC=Regression Coefficients, 95 % CI (95 % Confidence Interval); p < 0.05 was considered as significant.

## 4 Discussion

Data from the present study suggest that around 50 % of HCWs working in a tertiary hospital in Bangladesh suffered from infection by the SARS-COV-2 virus during the 3rd wave of the pandemic in the country. As the subjects were vaccinated with at least two doses of vaccines with reputed mRNA-based brands and no inactivated vaccine brand (which is known to generate Anti-N-Ab positivity) was included, the infection may be truly be termed as a ‘breakthrough’ infection (BTI). The study was conducted during February–March 2022 and the month of January coincided with the peak time of the Omicron wave [13,14]. Accordingly, it may be assumed that the breakthrough infection was caused by the Omicron variant of SARS-COV-2 which was reported to be the dominant strain circulating very rapidly during that time in Bangladesh (12).

Host factors are the most important weapons in fighting any infection and humoral immunity (as assessed by the antibody, especially IgG) is central to this defense. Natural infection and vaccination both are complementary factors to develop immunity against infectious diseases. Humoral immunity is more effective against symptomatic/severe disease than versus infection; risk reduction norms (face masks, social distancing, PPE, etc.) are essentials to control the transmission of the virus in health care premises [13,15]. In a study on HCW highly vaccinated against BTI (though almost all mild/asymptomatic) during omicron were by far acquired outside hospital, where health protection norms were not enforced [13]. This highlight also the importance of training HCWs to observe health protection measures and PPE when at work [16]. In the case of COVID-19, natural infection was initially the only means to develop immunity against the SARS-COV-2 virus for about a year. A number of mutations generated a few variants of concern (VOC) within that period which complicated the situation further. Availability of vaccines from the beginning of 2021 transformed the scenario with induced immunity by vaccines playing a major role to control the pandemic [17,18]. During the Omicron wave, it was reported that a pre-omicron primary infection is reportedly less protective than an omicron primary infection [17]. Moreover, contemporary evidence recommended to treat those with a documented infection similarly to those fully vaccinated with high-quality vaccines [18]. The vaccines were, in almost all cases, targeted against the Spike protein (S-protein) of SARS-COV-2 and thus anti-Spike protein antibody levels could not discriminate antibodies induced by vaccination and natural infection. Antibody against the nucleocapsid protein (Anti-N-Ab) thus became one of the important tools [[9], [10], [11]] to determine the extent of reinfection (ie infection after a natural infection) and breakthrough infection (ie infection after vaccination) which became quite common all over the world due to emergence of the Omicron variant wave at the end of 2021. The Anti-N-Ab level has also been suggested to be used for estimating vaccine effectiveness [19] and also a rationale has been created for the clinical evaluation of nucleocapsid-based mAb therapies to treat COVID-19 [20].

Bangladesh was fairly successful in vaccinating a major portion of its population with good-quality vaccines by the middle of 2021. Special priority was given to the HCWs who are the front liners in the fight against the pandemic. Accordingly, nearly all of the HCWs in the hospital under the study have been found to be vaccinated with two doses. Although males and females did not differ significantly in their initial effective vaccination status, the male employees were found to receive the 2nd Dose earlier than the females. Since vaccinations were provided by the Government, free of cost, with equal opportunity to both genders, it is likely that delay in receiving 2nd dose by the females were due to reluctance only. The reluctance, however, was reversed in receiving the 3rd (1st booster) dose of the vaccine where males were significantly delayed as compared to females. In spite of fully free booster dose being available within the campus from the beginning of 2022 only 23 % females and 33 % males were found to receive the booster dose by the end of February. Bangladesh suffered from the 3rd wave of the Pandemic during the end of 2021 to the beginning of 2022. The isolation or quarantine steps during this period was practically not so stringent like the initial periods of the pandemic. This is reflected in the present study by around 50 % seroprevalence of Anti-N-Ab among the subjects indicating reinfection or breakthrough infection among them.

As revealed by the antibody positivity status and the S/C Index (as a surrogate marker of the Anti-N-Ab level), the risk factors and immune response for BTI seems to differ between males and females in the present study. Central obesity (with WHR as an indicator) shows an independent positive association with BTI but negative association with immune response among males but not among the females. Obesity may be considered as another pandemic coexisting with COVID-19 and it is now considered as an important covariate of the disease related to infection susceptibility as well as clinical outcome [21,22]. Irrespective of presence of comorbidities (which are also associated with the condition) obesity independently affects virus susceptibility and clinical outcome in COVID-19 [23]. In a study by Polack et al. [24] it was found that, in a vaccinated cohort, people at the two extremes of BMI distribution (very low and very high BMI) are at greater risk of hospitalization or death from COVID-19 than people within normal weight range. Obesity is a recognized risk factor for COVID-19 [25], and both BMI and WHR were higher in males than females in the present study. The proportions of HCWs with overweight and obesity were also higher (p = 0.001) in males as compared to females. The present data indicate that, irrespective of gender, body weight within ideal ranges helps to prevent breakthrough infection in COVID-19.

Adequate coverage by a booster dose is another determinant of infection as well as level of immune response among this population. In the present study, the significantly earlier vaccination with booster dose (Table 1) may be another protective tool for the females against BTI. It was possible to observe that the 1st booster dose of the vaccine shows a definite trend of facilitation of the generation of natural humoral immunity among the study subjects. The findings are broadly in line with a Canadian study on more than 150,000 HCWs which provided evidence that 2 doses of vaccine strengthen humoral immunity after previous COVID-19 infection, whereas the booster dose did improve it further [17]. In a study on Croatian Healthcare Workers and infected hospitalized patients it has been shown that critically ill COVID-19 patients had higher levels of anti-Spike and anti-Nucleocapsid antibodies compared to HCWs [26]. It was encouraging to observe that, in the present study, none of the subjects suffered from severe forms of the disease as indicated by the nonrequirement of oxygen or ICU (data not shown) indicating that vaccination and/or previous infection provides good protection against the initial subvariant of the Omicron variant of COVID-19. Accordingly, vaccination against the virus must be encouraged among the community.

The study has several strengths including effective vaccination by brand products, reliable records in the hospital and detection of BTI by seroprevalence of the anti-nucleocapsid antibody. However, there are several weakness of the study which includes no concurrent rtPCR test data, absence of a reference group with no vaccination, and also inadequate number of subjects for multivariate analysis involving other covariates. Also, job tasks and hospital wards were not segregated in the analysis (again due to limitations in sample sizes) which have been included among the main determinants of COVID-19 infection [13,14] since patient-facing tasks increase the risk of BTI.

## 5 Conclusions

Data from the present study indicate that more than 50 % of the vaccinated hospital-based Health Care Workers in Bangladesh suffered from breakthrough infection during the winter of 2022 when the Omicron wave (the 3rd wave) of COVID-19 was at its peak. The data also show that overweight and obesity (with BMI and WHR as markers) are major host-related risk factors underlying breakthrough infection. Inadequate coverage by a booster dose seems to be another determinant of breakthrough infection as well as the level of immune response in this population.

## Ethics statement

The study was approved by the Ethical Review Committee of the Pothikrit Institute of Health Studies [Ref: PIHS/ IRRB/ LOA/ 12(3); Date: 31 August 2021]. Written Consent was obtained by all participants. Patients hospitalized for reasons other than COVID-19 and HCWs refusing to participate were excluded from the present study. No HCW was required to be excluded for such reasons.

## Additional information

No additional information is available for this paper.

## Data availability statement

Data associated with this study will be available on request.

## Funding statement

The research activities in this study was supported by the 10.13039/100030906Bangladesh Medical Research Council through 4th HPNSP Research Grant (2020-21/306).

## CRediT authorship contribution statement

**Masfida Akhter:** Writing – review & editing, Writing – original draft, Supervision, Project administration, Methodology, Investigation, Funding acquisition, Formal analysis, Data curation, Conceptualization. **Suman Kumar Roy:** Writing – original draft, Formal analysis, Data curation. **Abul Khair:** Writing – original draft, Supervision, Formal analysis. **Md Rabiul Karim:** Writing – original draft, Investigation, Formal analysis. **Ummel Khare Fatema Khan Mojlish:** Writing – original draft, Investigation, Formal analysis. **Minhaj Uddin Ahmed:** Writing – original draft, Formal analysis, Conceptualization. **Liaquat Ali:** Writing – review & editing, Writing – original draft, Supervision, Formal analysis, Conceptualization.

## Declaration of competing interest

The authors declare the following financial interests/personal relationships which may be considered as potential competing interests:Masfida Akhter reports financial support was provided by 10.13039/100030906Bangladesh Medical Research Council. If there are other authors, they declare that they have no known competing financial interests or personal relationships that could have appeared to influence the work reported in this paper.
