# Supplementary material for: SARS-COV-2 breakthrough infection and its covariates among healthcare providers of a hospital in Bangladesh during the omicron wave
Source: Heliyon. 2024 Sep 2;10(17):e37287. doi: 10.1016/j.heliyon.2024.e37287 (PMC11409073; doi:10.1016/j.heliyon.2024.e37287)
Supplement: Multimedia component 1 [file mmc1.pdf]

SARS-COV-2 BREAKTHROUGH INFECTION AND ITS COVARIATES AMONG HEALTHCARE PROVIDERS OF A HOSPITAL IN BANGLADESH DURING THE OMICRON WAVE

Place of the study: Narayanganj 300 Bed Hospital, Narayanganj/

তথ্য সংগ্রহের স্থান : নারায়নগঞ্জ ৩০০ শয্যা বিশিষ্ট হাসপাতাল, নারায়নগঞ্জ

Data collection Date/	Data collector's name/	Code of the study subject/
তথ্য সংগ্রহের তারিখ :	তথ্য সংগ্রহকারীর নাম :	তথ্য প্রদানকারীর কোড নম্বর :

(A) General information of the study subject /তথ্য প্রদানকারীর সাধারণ তথ্যাদি :

Name/ নামঃ

Age / বয়স:

Gender / পুরুষ / মহিলা:

Contact number/ ফোন নাম্বারঃ

Alternative contact number / বিকল্প ফোন নাম্বার:

E-mail/ ই-মেইলঃ

Designation/ পদবী :

(B) Clinical and anthropometric information / ক্লিনিক্যাল ও এন্থ্রোপোমেট্রিক তথ্যাদিঃ

Weight (Kg)/	Height(cm)/	BMI(kg/m^2)/
ওজনঃ	উচ্চতাঃ	বিএমআই :
Waist (cm):	Hip (cm):	Waist-Hip ratio
ওয়েস্ট :	হিপ :	ওয়েস্ট-হিপ রেশিও :

1. Do you have any chronic diseases/আপনার কি কোন পুরাতন/দীর্ঘস্থায়ী রোগ আছে?

a) yes /হ্যাঁ b) no/ না c) don't know/ জানা নেই

2. If the answer is yes, which of the following diseases?/ উত্তর হ্যাঁ হলে, কোন রোগটি ছিল?

a) Diabetes/ ডায়বেটিস; b) Hypertension /উচ্চ রক্তচাপ; c) COPD/ শ্বাসকষ্ট জনিত রোগ ; d) Renal diseases/ কিডনী জনিত রোগ; e) Cancer/ ক্যান্সার ; f) Asthma/ অ্যাজমা; g) Others/ অন্যান্য

(C) Information of the study subjects from March, 2020 to November, 2021/ মার্চ ২০২০ থেকে নভেম্বর ২০২১ পর্যন্ত তথ্যাদি :

3. Did you suffer from COVID symptoms anytime?/ এ সময়ের মধ্যে কি কোভিড এর উপসর্গ দেখা দিয়েছে?

a) yes / হ্যাঁ b) no / না

4. If yes, select from the followings/ উত্তর হ্যাঁ হলে, কী কী উপসর্গ ছিল?

a) Fever/ জ্বর ; b) Cough/ কাশি; c) Short breathing/ শ্বাস কষ্ট ; d) Throat ache/ গলা ব্যাথ;া e) Muscle pain/ শরীর ব্যাথা; f) Diarrhea/ ডায়রিয়া; g) Head ache/ মাথা ব্যাথা; h) No taste and smell/ স্বাদ ও গন্ধ না পাওয়া ;i) Conjunctivitis /চোখ ওঠা ; j) Fingers getting colorless/ হাত বা পায়ের আঙুল বর্ণহীন হয়ে যাওয়া; k) Others/ অন্যান্য

5. Did you have any test for COVID? / কোভিড নির্ণয়ের পরীক্ষা করেছিলেন কি?

a) yes/ হ্যাঁ b) no/ না

6. If yes, which test from the list?/ উত্তর হ্যাঁ হলে, কী পরীক্ষা করেছেন ?

a) rtPCR/ আরটিপিসিআর b)Antigen/ অ্যান্টিজেন

7. Was the report positive ?/ রিপোর্ট কি পজেটিভ হয়েছিল

a)Yes /হ্যাঁ b) no /না

8.Whether any need for hospitalization/ কোভিড আক্রান্ত হওয়ার পর কি হাসপাতালে ভর্তির প্রয়োজন হয়েছিল?

a)Yes / হ্যাঁ b) no / না

9. Whether any need for oxygen supplimentation কোভিড আক্রান্ত হওয়ার পরে কি অক্সিজেন দেওয়ার প্রয়োজন হয়েছিল?

a)Yes / হ্যাঁ b) no / না

10. Whether any need for ICU support?/কোভিড আক্রান্ত হওয়ার পর কি আইসিইউ তে ভর্তির প্রয়োজন হয়েছিল?

a)Yes / হ্যাঁ b) no / না

(D) Information after 30 Nov, 2021/ ৩০ নভেম্বর, ২০২১ পরবর্তী তথ্যসমূহ :

11. Did you get/have any symptom of COVID for the last 3 months?/গত ৩ মাসে কি কোভিড এর উপসর্গ দেখা দিয়েছে?

a) Yes / হ্যাঁ b) no / না

12. If yes, select from the followings/ উত্তর হ্যাঁ হলে, কী কী উপসর্গ ছিল?

a) Fever/ জ্বর ; b) Cough/ কাশি; c) Short breathing/ শ্বাস কষ্ট ; d) Throat ache/ গলা ব্যাথ;া e) Muscle pain/ শরীর ব্যাথা; f) Diarrhea/ ডায়রিয়া; g) Head ache/ মাথা ব্যাথা; h) No taste and smell/ স্বাদ ও গন্ধ না পাওয়া ;i) Conjunctivitis /চোখ ওঠা ; j) Fingers getting colorless/ হাত বা পায়ের আঙুল বর্ণহীন হয়ে যাওয়া; k) Others/ অন্যান্য

13. Did you have any test for COVID?/ কোভিড নির্ণয়ের পরীক্ষা করেছিলেন কি?

a) yes/ হ্যাঁ b) no/ না

14. If yes, which test from the list?/ উত্তর হ্যাঁ হলে, কী পরীক্ষা করেছেন ?

a) rtPCR/ আরটিপিসিআর b)Antigen/ অ্যান্টিজেন

15. Was the report positive ?/ রিপোর্ট কি পজেটিভ হয়েছিল?

a)Yes /হ্যাঁ b) no /না

16.Whether any need for hospitalization/ কোভিড আক্রান্ত হওয়ার পর কি হাসপাতালে ভর্তির প্রয়োজন হয়েছিল?

a)yes / হ্যাঁ b) no / না

17. Whether any need for oxygen supplimentation কোভিড আক্রান্ত হওয়ার পরে কি অক্সিজেন দেওয়ার প্রয়োজন হয়েছিল?
a)yes / হ্যাঁ b) no / না

18. Whether any need for ICU support?/কোভিড আক্রান্ত হওয়ার পর কি আইসিইউ তে ভর্তির প্রয়োজন হয়েছিল?

a) yes / হ্যাঁ b) no / না

19. Vaccinatin related information /ভ্যাক্সিনেশন সংক্রান্ত তথ্যসমূহ ঃ

20. Did you get vaccine? / আপনি কি ভ্যাক্সিন নিয়েছেন?

a) yes/ হ্যাঁ b)no/ না

21. If the Answer is yes, please give the related information below/ উত্তর হ্যাঁ হলে,ভ্যাক্সিন গ্রহণ সম্পর্কিত তথ্য ঃ

Doses/ ডোজ সংখ্যা	Vaccination date/ গ্রহণের তারিখ	Name of the manufacturer/ ভ্যাক্সিন প্রস্তুতকারী প্রতিষ্ঠান এর নাম
1		
2		
3		
